# Postpartum Haemorrhage and Carboprost for Its Prevention: A Narrative Review

**DOI:** 10.7759/cureus.62875

**Published:** 2024-06-21

**Authors:** Saloni, Manjusha Agrawal

**Affiliations:** 1 Obstetrics and Gynecology, Jawaharlal Nehru Medical College, Datta Meghe Institute of Higher Education and Research, Wardha, IND

**Keywords:** uterotonics, bleeding in pregnancy, active management of third stage of labour, third stage of labour, haemorrhage in pregnancy

## Abstract

The most frequent and harmful side effect of childbirth is obstetric haemorrhage. Postpartum haemorrhage (PPH) remains the primary cause of maternal mortality worldwide. Most PPH-related deaths take place in the first 24 hours of life. It is commonly believed that prompt diagnosis and treatment could avert the majority of PPH-related deaths. The rapid transition of haemorrhage from the remunerated to the decompensated stage is frequently overlooked. For this reason, anticipation, early detection, and management are crucial to reducing the risk of severe PPH (SPPH) or improving its clinical outcomes. Third-stage labour is a high-risk period for PPH. Active management of PPH is an effective intervention to lessen the incidence of PPH and has been promoted as a means of lowering fatality rates. Currently, prostaglandins (PGs) are advised as a second-line uterotonic medication. Strong uterotonic drugs such as carboprost tromethamine play a physiological role in human parturition, helping to birth the fetus and controlling PPH. Prostaglandins have a major effect on uterine tone, which minimizes blood loss. Their discovery, together with the use of their counterparts as uterotonics, has improved PPH management. In order to assist healthcare professionals in managing PPH promptly and minimizing adverse effects on both the mother and the newborn, this review will describe the causes of the disorder, the strategies that have been tried to treat it, and the role that carboprost plays in preventing it.

## Introduction and background

Pregnancy- and childbirth-related deaths are known as maternal deaths. These deaths are seen as preventable and rank among the major public health issues in developing and impoverished countries. The frequent causes of maternal death are infection, eclampsia, obstructed labour, unsafe abortions, and postpartum haemorrhage (PPH). The loss of blood of more than 500 millilitres (mL) during delivery via the vagina and 1000 mL after caesarean delivery is generally referred to as PPH. However, there are differences in definitions, and the diagnosis of PPH is frequently based on imprecise estimations of blood loss. Furthermore, the usual blood loss during birth often surpasses 500 or 1000 mL, and the normal increases in plasma volume that happen during pregnancy may conceal signs of haemorrhage or shock from blood loss [1,2].

In contemporary obstetrics, routine active management of the third stage of labour (AMTSL) may be crucial in lowering maternal mortality and morbidity from PPH. In order to prevent PPH, uterotonics such as oxytocin, methylergometrine, and 15-(S)-15-methyl PGF2α are employed. According to studies, there are significant differences in how third-stage labour is managed. It has been demonstrated that uterotonic treatment reduces PPH incidence by 40%. However, there are adverse consequences linked to it, such as pulmonary oedema, myocardial infarction, vomiting, hypertension, and shivering diarrhoea. An intramuscular form of PGF2α mimic is carboprost (CP) tromethamine [3]. It has been shown to be 84%-96% successful in treating uterine atony-related persistent haemorrhage. But, since its debut, there have been few studies evaluating its efficacy in treating and preventing PPH, and only one particularly looking at its application after caesarean delivery [4].

## Review

Epidemiology

According to data from the World Health Organization (WHO), PPH is the most common cause of illness and mortality among mothers worldwide, accounting for 25% of all maternal fatalities. In India, the incidence of PPH is 6% after a C-section and 2%-4% after a vaginal delivery. It is a significant factor in India's 19.9% maternal death rate. Approximately 70,000 maternal deaths occur worldwide every year, predominantly in low- and middle-income nations, as a result of the approximately 14 million women who suffer from PPH. This is the equivalent of one fatality every six minutes. PPH-related deaths are mostly avoidable and have all but disappeared in high-income nations (HICs) [5,6].

Risk factors for PPH

PPH cases are increasing because of factors such as women's advanced age at childbearing, increased body mass index (BMI), large birth weight, inadequate prenatal care, shortage of trained birth attendants, delayed medical attention seeking, and restricted access to emergency obstetric services [7]. Of the various causes of PPH, uterine atony is the most prevalent. At least 75% of PPH cases and 1 in 40 complicated births in the United States are caused by the uterus not contracting well after delivery. While prolonged labour may result in uterine tiredness or inadequate contractions, which in turn produce postpartum uterine atony, quick labour is thought to be connected with powerful contractions that tire out the uterus. Atony can also be brought on by increased or induced labour, which all contribute to the development of PPH [8]. In many parts of the world, fetal macrosomia (FM), or gestational weight ≥ 4,000 g, is acknowledged as a risk factor for PPH, being mostly linked to dyslipidaemia, a history of a macrosomic fetus, diabetes, obesity before becoming pregnant, an increase in weight during pregnancy, abnormal fasting and postprandial glucose levels, and post-term pregnancy. The results of the meta-analysis indicated that FM raises the possibility of PPH in expectant mothers. Only three investigations investigated the relationship between severe PPH and FM, and despite significant heterogeneity, the meta-analysis did not find any correlation between both clinical disorders [9].

Genital Tract Trauma

If genital tract injuries are not detected right away, it might result in bleeding and a significant volume of PPH. More than 85% of women who give birth vaginally will experience some perineal trauma, and between 60% and 70% of them will require suturing. To detect any injuries to the cervix, vagina, or perineum, a thorough examination of the genital tract should be carried out. A competent obstetrician should treat upper vaginal or cervical rips since there is a chance that the surrounding structures could be harmed [10]. PPH can be directly caused by coagulation problems. Furthermore, severe bleeding can cause coagulation factors to be consumed, which might worsen dilutional coagulopathy following volume resuscitation and cause PPH to worsen. PPH may result from widespread intravascular coagulopathy, which includes sepsis, amniotic fluid embolism, significant blood loss or transfusion, intrauterine fetal death with protracted retention of a dead fetus, and placental abruption. Excessive blood loss after giving birth is also linked to other coagulation diseases such as thrombocytopenia, von Willebrand disease, and anticoagulant medication [11]. Research has indicated a connection between advanced maternal age and PPH, but there is currently insufficient agreement on this topic. One study found that mothers over 35 years old had a higher chance of developing SPPH [12].

According to Sheen et al., women who are older than 45 years of age are more likely to experience a variety of negative outcomes during their hospital stay after giving birth, including PPH. However, a meta-analysis showed no relationship between maternal age (more than equal to 35) and PPH. Furthermore, another study revealed that growing older has a preventive effect on PPH [13]. Maternal age < 18 years was associated with increased SPPH, although older mother age (≥35y) increased the incidence of severe PPH in univariate analysis, according to the results of another study. The adjusted ratio had a wide confidence interval even though among the patients with SPPH, there was only one woman (0.20%) whose maternal age was less than 18 years; among the controls, it was 0.06% [14].

The risk of severe PPH (SPPH) was 4.94 times higher in women with a history of PPH. Similarly, an Australian study found that 28% of medical audits had recurrences [15]. According to a Swedish study, the recurrence of PPH may be explained by genetic and environmental variables [16]. Consistent with earlier research, women who conceived by IVF had a higher risk of developing SPPH. Placental adherence increased in a group following the use of assisted reproductive technology, according to Tang et al. [17]. According to the study, a C-section was linked to a higher risk of SPPH in the univariate analysis. On the other hand, the multivariate model showed that women who had caesarean deliveries had a 43% lower incidence of SPPH. Most earlier research findings were at odds with the beneficial benefits of C-sections. But as compared to vaginal births, a few studies have found that C-sections protect against PPH. Consistent with earlier research, women who experienced placenta previa, placental abruption, or placenta accreta spectrum (PAS) had a considerably higher risk of SPPH. PPH following blood transfusion and PPH after hysterectomy are examples of severe cases of PPH when placenta-related variables played a key role [18].

Strategies to reduce morbidity and mortality from PPH

PPH primarily manifests during the third stage of labour (TSL), and it can be avoided using AMTSL. Using uterotonics in the TSL before placenta delivery is the most efficient course of action to prevent PPH. The best medication is an injectable uterotonic, although if one is not available, oral misoprostol may be used instead. Initiatives to support anaemia correction may be required, as up to 25% of pregnant women suffer from prenatal anaemia. Some professional associations have released PPH guidelines, which suggest that the therapeutic objective be haemoglobin (Hb) level of more than 8 g/dL. AMTSL is linked to a significant decrease in the incidence of PPH as compared to expectant management [19]. Proactive uterine massage, controlled cord traction (CCT), and prophylactic oxytocin are helpful methods during the TSL. If bleeding occurs and the placenta remains in place, it should be manually removed as quickly as feasible. It is well-recognized that macrosomia causes the uterus to become over-dense, which is linked to uterine atony [20].

Evidence-Based Interventions to Prevent PPH

AMTSL is a term used to describe a series of treatments designed to avoid PPH [21]. AMTSL entails giving all women uterotonics, preferably within one minute of delivery, CCT to promote placental delivery, uterine massage to initiate uterine contraction, and tonus assessment every 15 minutes for the first two hours after delivery to detect uterine atony early [22,23]. The WHO advises that competent birth attendants and medical professionals with pregnancy and delivery management training should perform AMTSL [24].

Trained community health workers or professional providers can apply evidence-based therapies that lower the incidence of PPH and are appropriate for low-resource settings. These are women who give birth without the assistance of a competent clinician using misoprostol or AMTSL. For a long time, uterotonics have been used to treat uterine atony and lessen the amount of blood lost after childbirth. They also cause uterine contractions. One of the most crucial measures to avoid PPH is the use of a uterotonic medication as soon as the baby is delivered. AMTSL comprises three primary steps: delivering the placenta to the uterus with CCT and counter-traction; injecting a uterotonic drug (ideally oxytocin) within a minute of the baby's delivery; and massage of the uterus following placenta delivery, together with uterine palpation to determine whether massage therapy is necessary for the two hours that follow placenta delivery. In the absence of a qualified practitioner who can perform AMTSL, recent WHO guidelines authorize the administration of misoprostol by a health professional trained in its use for the prevention of PPH. Because of their affordability, efficiency, and safety, misoprostol pills are the best option for treating PPH in home births in environments with limited resources [25].

A Cochrane study evaluated the impact of preventive oxytocin administered during the TSL on PPH. A total of 20 randomized controlled trials (RCTs) with 10,806 women were included in the review. When compared to a placebo, prophylactic oxytocin halved the risk of PPH. Compared to ergot alkaloids, it resulted in a 25% lower risk of PPH. When oxytocin and ergometrine were taken together, the risk of PPH was not significantly different from when ergot alkaloids were taken alone. Ergot alkaloids were not as well tolerated as oxytocin [6].

Other Interventions

Uterine massage: There is little to no clear evidence supporting the effectiveness of uterine massage in preventing PPH. Information from two RCTs with 1,491 participants that looked at the advantages of uterine massage before, following, or concurrently with placenta delivery was assessed by a Cochrane review. Regardless of when the massage was started, the intervention and control groups did not significantly differ in the amount of uterine blood lost. When it comes to preventing PPH in women who have received prophylactic oxytocin, the WHO does not advise prolonged uterine massage. For all women, however, it is recommended to detect uterine atony, a failure of the uterus to contract adequately as soon as possible after giving birth [26].

Cord clamping early versus late: A Cochrane analysis evaluated the impact on maternal and newborn outcomes of early versus late cord clamping following delivery. Included in the review were 15 trials with 3,911 mother and child pairs carried out in low- and middle-income countries (LMICs) and high-income countries (HICs). PPH and severe PPH in the mothers did not significantly differ between the early and late cord clamping groups. In contrast, early and late cord clamping enhanced infants' early Hb concentrations and iron reserves; hence, to improve baby outcomes, the WHO recommends late cord clamping [27].

Controlled cord traction: There have been two significant CCT trials, one involving 23,861 women in eight LMICs and the other involving 4,013 women in France. The findings of these trials imply that CCT, when used to control the TSL, has no discernible clinical impact on the prevalence of PPH. The WHO recommends that CCT should be performed by trained birth attendants [28].

Management of PPH

An integrated team effort combining effective communication between an anesthesiologist, a haematologist/blood bank, and an interventional radiologist is necessary for PPH therapy, along with an obstetrician and perinatologist. Precise blood loss evaluation, keeping an eye on maternal symptoms, replenishing fluids, blood, and blood products, and halting the bleeding source all depend on this [29].

Blood Loss Evaluation

Accurate blood loss measurement is crucial for determining the extent of bleeding and evaluating the patient's reaction to bleeding. A reference guide with pictures to aid in the visual computation of blood loss declares that one may estimate blood loss using the following items: a soaked, soiled sanitary towel, a tiny and large soaked swab, and a full kidney dish. On the other hand, misdiagnosis rates in visual estimations range from 35% to 50%. Therefore, since estimated blood loss (EBL) is always extremely difficult and delayed, quantitative testing is recommended instead of EBL [30].

Management Algorithm

In addition to uterotonics, non-surgical methods, such as balloon tamponade, surgical procedures like compression sutures, ovarian-uterine artery ligations, ligation of the iliac artery (Internal), and/or hysterectomy peripartum, can be employed in the management of PPH.

Fluids in Case of Bleeding

When bleeding, it is advised to restore lost extracellular fluid with isotonic crystalloids as soon as possible using a protocol-based approach. Compared to crystalloids, the infusion of colloids results in haemodynamic stabilization with less tissue oedema. In cases of severe bleeding, aggressive volume loading may exacerbate dilutional coagulopathy. Thus, it is crucial to minimize hypervolaemia and dilution of coagulation factors that could worsen coagulopathy by replacing blood with a restricted amount (1-2 mL crystalloid for every 1 mL blood loss) [31].

Carboprost

The potent uterotonic agent PGF2α plays a physiological role in human parturition, controlling PPH as well as facilitating foetal birth. Since more than three decades ago, CP tromethamine, a more powerful counterpart of 15-(S)-15-methyl PGF2α, has been in use. This is longer-acting and more effective than natural PGF2α. Thus, prostaglandins are the class of uterine-stimulating medicines with the fewest adverse effects that are used to manage TSL and avoid postpartum haemorrhage. For treating PPH, it is given intramuscularly every 15 minutes up to a maximum of eight times. More than 10% of women who receive treatment report experiencing the most frequent adverse effects, which include nausea, vomiting, and diarrhoea. Asthmatics must exercise caution since it can result in bronchospasm [32].

Promising results have been found in the few trials that have looked at the effectiveness of a prophylactic dosage of CP in PPH prevention. To AMTSL, intramuscular methyl ergometrine (ME), misoprostol sublingual, and intramuscular CP and ME were studied by Vaid et al. as preventive measures. They discovered that all three medications were equally effective in preventing PPH, although the most common side effect of CP was diarrhoea [33]. Abdel-Aleem et al. evaluated the extent of the TSL and the blood loss (mean) between 150 women treated with CP and ME and found that the former was substantially shorter [34].

According to Sunil Kumar et al.'s research, 97% of patients experienced satisfactory prevention of PPH using CP, a potent uterotonic. Compared to oxytocin, only four patients required additional uterotonic, which caused 21 patients to require extra medication to stop excessive bleeding. When it came to reducing PPH in high-risk patients having C-sections, Bai et al. found that CP was superior to oxytocin in this regard. In their investigation, vomiting was relatively common among patients who got CP [35,36]. When used in a therapeutic dose of 250 μg, CP has been shown to be 84%-66% effective in treating PPH and may prevent the need for surgical procedures. However, people who receive the recommended dosage of CP inevitably experience nausea, vomiting, fever, and asthma. In addition, patients may get heat flashes, perspire, and get agitated. However, at the preventive dose of 125 μg, these side effects are significantly reduced [37].

In a comparative trial of various uterotonics for atonic PPH in high-risk patients, Reddy and Shenoy discovered that the average length of the TSL was 2.44 minutes for the ME group and 2.33 minutes for the CP group. With ME, the mean blood loss was 202±84 mL, while with CP, it was 127±97 mL [38]. In a related study on the impact of uterotonics in AMTSL, Bhattacharya et al. discovered that the group receiving ME had a mean TSL duration of 8.06 minutes, whereas the group receiving CP had a mean duration of 4.8 minutes [39].

The durations for the CP and ME groups in a different study were 2.63 minutes and 3.6 minutes, respectively [40]. In the Singh et al. trial, adverse effects included vomiting (number 1) and diarrhoea (number 1) in two (3%) of the ME group and nausea in eight (12.4%) of the CP group (number 2) [41].

In research on women who had vaginal births after 28 weeks of amenorrhoea and had one or more haemorrhagic risk factors (HRF), the participants were split into two groups: one group received oxytocin alone, and the other received oxytocin along with CP. They discovered that for the HRF of multiparity and uterine overdistension, blood loss was reduced by oxytocin and then CP (p=0.003; p=0.00001). They concluded that in patients with HRF, particularly those with multiparity and uterine overdistension, the synergy of two uterotonics, oxytocin and CP, helps to reduce postpartum bleeding. For parturients at higher risk, adding CP is a good substitute for reducing PPH [42]. When Lamont et al. evaluated the effectiveness of CP and syntometrine (SM) in preventing PPH, they found that both medications prevented PPH as well. Compared to 0.8% of patients who received SM, 21% of patients who took CP experienced diarrhoea. While major adverse effects are rare and self-limited, the previously mentioned potential drawbacks still exist [43].

CP reduces the TSL and reduces blood loss when compared to standard uterotonics; nevertheless, it raises the possibility of diarrhoea, vomiting, and stomach pain. However, CP can bring on bronchospasms. Desaturation of arterial Hb oxygen can be brought on by intrapulmonary shunting in conjunction with increases in systemic and pulmonary vascular resistance. Individuals with asthma are more vulnerable to these issues, yet some occurrences of bronchospasm in non-asthma individuals have been documented. It is important to remember that PGF2α is an endogenous substance that has a role in both disease and physiology. Several experimental and clinical investigations have demonstrated the connection between PGF2α and severe acute or chronic inflammatory diseases, which is an increasing risk factor for diabetes, septic shock, atherosclerosis, and a host of other conditions [44-47]. CP should be administered cautiously in individuals with the aforementioned conditions, particularly if repeated injections are required [48].

CP has a few disadvantages that should be taken into account. Firstly, it is a costly medication. Secondly, not all locations may have the prophylactic dose readily available. Lastly, the drug must be refrigerated between 2-4 °C. It is a costly therapy that needs to be kept cold, making it challenging to use in environments with limited resources.

## Conclusions

Even though PPH is one of the primary preventable causes of maternal death, disparities in women's access to healthcare and the kind of care they receive account for the fact that PPH remains the most common cause of maternal death. TSL is when there is the highest risk of PPH due to potential irregular contractions of the uterus following childbirth. AMTSL is a highly effective technique for lowering the death rate of mothers. It provides a significant therapeutic advantage in reducing maternal issues with minimal risk, and it ought to be adopted as routine treatment at a minimal cost. Based on the above-described studies, CP is more effective than oxytocin at preventing PPH in high-risk recipients of CD. However, there were side effects associated with it, and the medication was well taken. For this patient type, CP might be considered a suitable drug for the AMTSL. Programmes to identify and stop early risk factors that could condition the occurrence of PPH and other maternal problems need to be improved and put into action.
